# Supplementary material for: Low Antenatal Care Number of Consultations Is Associated with Gestational Weight Gain and Birth Weight of Offspring of Teenage Mothers: A Study Based on Colombian and Mexican Cohorts
Source: Nutrients. 2024 Oct 31;16(21):3726. doi: 10.3390/nu16213726 (PMC11547604; doi:10.3390/nu16213726)
Supplement: Supplementary file 1 [file nutrients-16-03726-s001.zip › nutrients-3271703-supplementary.pdf]

Supplementary Table S1. Inadequate (insufficient-excessive) gestational weight gain (GWG) according to sociodemographic characteristics of pregnant adolescents (%).

Variable	GWG-Colombia		p*	GWG-Mexico		p*
	Insufficient	Excessive		Insufficient	Excessive	
Chronological age (years)						
≤15	67	11	0.953	38	35	0.154
≥16	68	12		34	30	
Marital status before and during pregnancy (%)						
Cohabiting with father's baby before	25	27	0.828	39	30	0.789
Cohabiting with father's baby during	31	50	0.336	32	37	0.070
Socioeconomic status (%)						
Middle low	68	16	0.484	37	34	0.937
Low	65	10		34	34	
Very low	73	13		38	30	
Family structure (%)						
Nuclear	75	10	0.783	35	34	0.862
Nuclear with partner	86	0		56	11	
Extended combined	67	13		36	32	
Single-parent with partner	61	15		36	33	
Educational lag						
Yes	66	13	0.642	36	28	0.200
No	73	9		36	36	
Economic provider (%)						
Partner						
Yes	74	8	0.179	46	33	0.223
No	63	16		34	33	
Herself						
Yes	67	0	0.527	0	0	-
No	68	12		36	33	
Parents						
Yes	64	12	0.168	34	34	0.236
No	75	13		45	31	
Extended family						
Yes	61	13	0.647	42	26	0.769
No	69	12		35	34	

* p-value by Pearson Chi^2. pBMI: Pregestational body mass index. Percentages estimated by rows.

Supplementary Table S2. Inadequate (insufficient-excessive) gestational weight gain (GWG) among pregnant adolescents according to their general characteristics (%)

Variable	GWG Colombians		p*	GWG Mexicans		p*
	Insufficient n=200	Excessive n=35		Insufficient n=142	Excessive n=131	
pBMI (%)						
Underweight	57	14	0.601	47	18	**<0.001**
Normal	70	11		40	25	
Overweight	54	23		16	70	
Obesity	33	33		33	58	
Antenatal care initiation by trimester (%)						
1st (1-13 weeks)	69	11	0.916	38	27	0.756
2nd (14-26 weeks)	67	15		36	35	
3rd (27-40 weeks)	64	9		35	31	
Number of antenatal care consultations						
≤7 inadequate	39	21	**0.007**	35	35	**0.041**
≥8 adequate	72	11		39	20	
Gynecological age (years)						
≤3	74	7	0.429	37	33	0.748
≥4	66	14		35	32	
Gestational age at birth						
Term	68	13	0.318	38	31	**0.040**
Preterm	63	0		19	49	
Sex						
Girls	72	13	0.292	37	32	0.896
Boys	64	12		35	34	
Delivery						
Vaginal birth	67	13	0.781	39	34	0.112
Cesarean section	73	10		32	32	

*Pearson Chi^2, NB: newborn. Percentages estimated by rows. Adequate GWG was not included in the table.

Supplementary Table S3. Newborn weight according to clinical and sociodemographic characteristics of pregnant adolescents

Variables	Colombia n=294		p	Mexico n=396		P
	SGA n=47	NSGA n=247		SGA n=92	NSGA n=304	
Underweight	75	25	0.485	47	53	**0.028**
Normal	82	18		76	24	
Overweight	93	7		81	19	
Obesity	100	0		83	17	
GWG						
Insufficient	18	82	0.177	27	73	0.783
Adequate	5	95		24	76	
Excessive	18	82		25	75	
1st (1-13 weeks)	85	15	0.577	76	24	0.208
2nd (14-26 weeks)	80	20		78	22	
3rd (27-40 weeks)	79	21		67	33	
≤7 inadequate	21	79	**0.045**	28	72	**0.006**
≥8 adequate	6	94		89	11	
≤3	24	25	0.889	68	54	**0.014**
≥4	76	75		31	46	
Term	83	17	0.566	77	23	0.436
Preterm	77	23		71	29	
Girls	77	23	**0.029**	75	25	0.461
Boys	87	13		78	22	
Vaginal birth	83	17	0.951	77	23	0.682
Cesarean section	83	17		75	25	
≤15	91	9	0.119	78	22	0.394
≥16	81	19		75	25	
Cohabiting with father's baby before pregnancy	21	1	0.538	19	0	0.330
Cohabiting with father's baby during pregnancy	32	42	0.295	37	45	0.342
Middle low	79	21	0.749	76	24	0.832
Low	84	16		77	23	
Very low	84	16		73	27	
Nuclear	87	13	0.687	76	24	0.603
Uniparental	83	17		72	28	
Extended	79	21		82	18	
Educational lag (%)						

Yes	83	17	0.892	77	23	0.388
No	83	17		73	27	
Economic provider (%)						
Herself						
Yes	80	20	0.812	0	0	NA
No	83	17		76	24	
Partner						
Yes	87	13	0.085	84	16	0.257
No	79	21		75	25	
Parents						
Yes	83	17	0.750	76	24	0.794
No	82	18		77	23	
Extended family						
Yes	79	21	0.482	67	33	0.289
No	84	16		77	23	

*Pearson Chi^2; SGA: small for gestational age; NSGA: non-small for gestational age; NA: not applicable. Percentages estimated by rows.

Supplementary Table S4. C-section delivery according to clinical and sociodemographic characteristics of pregnant adolescents.

Variables	Colombia n=294			México n = 396		
	C-section 78%	Vaginal 22%	p	C-section 50%	Vaginal 50%	P
pBMI (%)						
Underweight	25	75	0.330	17	82	0.056
Normal	20	80		51	49	
Overweight	33	66		51	49	
Obesity	50	50		58	42	
GWG						
Insufficient	23	76	0.781	44	56	0.112
Adequate	19	81		57	43	
Excessive	18	82		48	51	
Antenatal care initiation by trimester (%)						
1st (1-13 weeks)	22	78	0.472	46	54	0.694
2nd (14-26 weeks)	23	77		51	49	
3rd (27-40 weeks)	10	89		52	48	
Number of antenatal care consultations						
≤7 inadequate	22	78	0.381	43	57	0.232
≥8 adequate	16	84		51	49	
Gynecological age (years)						
≤3	26	74	0.409	50	50	0.849
≥4	21	79		49	51	
Gestational age at birth						
Term	21	79	0.895	49	50	0.453
Preterm	23	77		55	45	
Sex						
Girls	21	79	0.907	52	48	0.329
Boys	22	78		47	53	
Chronological age (years)						
≤15	27	73	0.319	49	51	0.839
≥16	20	80		51	49	
Cohabiting with father's baby before pregnancy	24	76	0.579	58	42	0.128
Cohabiting with father's baby during pregnancy	19	81	0.406	50	50	0.965
Socioeconomic status (%)						
Middle low	19	81	0.764	47	53	0.544
Low	23	77		48	52	
Very low	21	79		56	44	

Family structure (%)						
Nuclear	20	80		54	46	
Uniparental	24	76	0.503	58	41	0.032
Extended	24	76		37	63	
Educational lag (%)						
Yes	19	81	0.104	47	53	0.458
No	29	71				
Economic provider (%)						
Herself						
Yes	20	80	0.900	0	0	NA
No	22	78		50	50	
Partner						
Yes	23	77	0.733	24	46	0.613
No	21	79		50	50	
Parents						
Yes	25	75	0.099	51	49	0.242
No	16	84		43	57	
Extended family						
Yes	26	74	0.429	24	76	0.014
No	21	79		52	48	

* Pearson Chi^2; SGA: small for gestational age; NSGA: non-small for gestational age; NA: not applicable. Percentages estimated by rows.
